# Clinical study on closed Kirschner wire prying reduction for Gartland Type IV supracondylar humeral fractures in children and analysis of typical cases

**DOI:** 10.3389/fsurg.2026.1774159

**Published:** 2026-02-23

**Authors:** Yunpeng Wu, Jingrong Wen, Weijun Hui, Jianglong Wang, Fangjun Yang, Xiaoming Qiu, Yunping Peng, Zhimin Yuan

**Affiliations:** 1 Department of Orthopedics, Gannan Prefecture People’s Hospital, Hezuo, Gansu, China; 2 Department of Orthopedics, Gansu Provincial Hospital of Traditional Chinese Medicine, Lanzhou, Gansu, China

**Keywords:** children, closed fracture reduction, Gartland Type IV, reduction, supracondylar humeral fracture

## Abstract

**Objective:**

To explore the feasibility, safety and clinical efficacy of closed Kirschner wire prying reduction technique in the treatment of Gartland Type IV supracondylar humeral fractures in children.

**Methods:**

A retrospective analysis was conducted on the clinical data of 24 children with intraoperatively confirmed Gartland Type IV supracondylar humeral fractures admitted to Gannan Prefecture People's Hospital from January 2018 to May 2025. After the failure of closed manual reduction, all children underwent closed reduction using percutaneous Kirschner wire prying technique, followed by cross or double lateral Kirschner wire fixation. The operation time, reduction success rate, occurrence of complications and postoperative functional recovery were collected. In addition, 2 representative cases were selected to display typical imaging data.

**Results:**

Among the 24 children, 22 achieved successful reduction without open surgery; the average operation time was 42 min. One child had mild re-displacement after surgery, and one child had transient ulnar nerve palsy. All cases achieved good fracture healing, and the excellent and good rate of Flynn score at the last follow-up was 86.4%. The postoperative imaging of typical cases showed good alignment, and the elbow joint function was completely restored.

**Conclusion:**

For children with Gartland Type IV supracondylar humeral fractures, closed Kirschner wire prying reduction technique is a safe and effective minimally invasive reduction method, which may effectively reduce the need for open reduction, and has good clinical application value.

## 1 Introduction

Supracondylar humeral fracture in children is the most common type of elbow fracture, accounting for approximately 15% of all elbow fractures in children. Among them, Gartland Type III fractures usually require surgical intervention due to their complete displacement. However, Gartland Type IV fracture is a more complex variant, characterized by multidirectional instability, usually detected intraoperatively, with complete tearing of the annular periosteal hinge around the fracture end leading to severe instability and high failure rate of closed reduction (1, 2). Traditionally, open reduction and internal fixation are mostly used for treatment.

Although open reduction can clearly expose nerves and blood vessels during the operation and effectively achieve reduction of difficult-to-reduce fractures, it causes great trauma, and complications such as joint stiffness, scar formation and myositis ossificans may occur after surgery. Therefore, in recent years, minimally invasive techniques have attracted increasing attention in the treatment of children's fractures. Among them, closed Kirschner wire prying reduction technique, as a minimally invasive reduction method combining image guidance and lever principle, has achieved good clinical results in the treatment of Gartland Type III fractures (3). And kirschner wire prying can effectively improve the success rate of closed reduction and reduce the rate of open reduction (4). However, systematic studies on Gartland Type IV fractures are still relatively scarce.

This study aims to evaluate the feasibility, safety and clinical efficacy of closed Kirschner wire prying reduction technique in the treatment of Gartland Type IV supracondylar humeral fractures in children. By retrospectively analyzing the clinical data of 24 children with Gartland Type IV fractures treated with this technique in our department, combined with detailed imaging data of 2 typical cases, the application value of this technique in terms of reduction success rate, complication control and functional recovery was explored, in order to provide a practical new minimally invasive reduction idea for clinical practice.

## 2 Materials and methods

### 2.1 Study design

This was a single-center retrospective study. The cases of children with intraoperatively confirmed Gartland Type IV supracondylar humeral fractures admitted to our department from January 2018 to May 2025 were collected. After the failure of conventional manual reduction, all children were treated with closed Kirschner wire prying reduction, followed by plaster fixation and regular follow-up.

This was a single-center retrospective observational study approved by the Ethics Committee of Gannan Prefecture People's Hospital (Approval No.: ZYY-2025-011. All procedures complied with the Declaration of Helsinki. Informed consent for treatment and study participation was obtained from the guardians of all children, and informed consent for image publication was separately obtained for typical cases.

### 2.2 Inclusion and exclusion criteria

Inclusion criteria: (1) Age ≤ 14 years old, with supracondylar humeral fracture; (2) Confirmed as Gartland Type IV (unstable in both flexion and extension directions) by intraoperative fluoroscopy; (3) Treated with closed Kirschner wire prying reduction and successfully received closed Kirschner wire internal fixation; (4) Follow-up for at least 6 months after surgery, with complete imaging and clinical data.

Exclusion criteria: (1) Multiple injuries or combined with other elbow fractures; (2) Obvious pre-operative neurovascular injury; (3) Converted to open reduction after reduction failure; (4) Incomplete follow-up data.

### 2.3 Surgical method

The child was fixed in a supine position under general anesthesia, with the affected limb abducted at 90°. Conventional closed manual reduction was attempted under fluoroscopy. If the reduction failed, Kirschner wire prying was used for auxiliary reduction: a Kirschner wire with a diameter of 2.0–2.5 mm was selected according to the age of the child, and inserted percutaneously at the distal end of the humerus, with the needle tip reaching approximately 1–2 cm distal to the fracture end at the level of the humeral epicondyles. Under the guidance of C-arm fluoroscopy, the Kirschner wire was used as a fulcrum, and the distal end of the fracture was reduced to the anatomical position by moderate prying. After the reduction was completed, cross or double lateral Kirschner wire fixation was performed immediately under the guidance of C-arm. After the operation, plaster slab external fixation was applied for 4 weeks, and functional rehabilitation training was conducted after the wire was removed.

### 2.4 Observation indicators

Reduction success rate: Successful closed prying reduction without the need for open surgery.

Operation time: From the start of closed reduction to the end of fixation of the last Kirschner wire.

Complications: Including fracture re-displacement, nerve injury, infection, etc.

Clinical efficacy evaluation: At the last follow-up, the elbow joint function was evaluated according to the Flynn criteria, including flexion-extension function, symmetry and complications.

### 2.5 Data analysis

Descriptive analysis was mainly used. The reduction success rate and complication rate were reported as percentages.

## 3 Results

### 3.1 General data

A total of 24 children were included in this study, all of whom were diagnosed with Gartland Type IV supracondylar humeral fractures by intraoperative fluoroscopy. There were 16 males and 8 females, with an average age of 8.2 ± 1.7 years (range: 4–14 years). The causes of injury included 16 cases of fall injury, 5 cases of horse-riding injury and 3 cases of traffic accident injury. Fracture side: 9 cases on the left side and 15 cases on the right side. After the failure of attempted closed manual reduction, all children underwent closed Kirschner wire prying reduction. Among them, 22 children successfully completed closed reduction and fixation through Kirschner wire prying, with a closed reduction success rate of 91.7%. Two cases were eventually converted to open reduction due to severe impaction of the fracture end and soft tissue interference. Kirschner wire fixation methods: 18 cases with cross-wire fixation and 6 cases with double lateral wire fixation. The average operation time was 42.5 ± 9.3 min, the intraoperative blood loss was extremely small (<5 mL), and no intraoperative neurovascular injury occurred. All children were given plaster slab fixation for 4 weeks after surgery. Explicitly, all patients recruited at the end of the study period (May 2025) had completed the full 6-month follow-up by the time of data lock, meeting the inclusion criteria. The post-operative follow-up period was 6–12 months (average: 8.4 months). During the follow-up period, no obvious fracture re-displacement or Kirschner wire loosening was found. One child had mild pin tract redness and swelling after surgery, which recovered after dressing change. No complications such as cubitus varus, cubitus valgus deformity or limited function were observed. The elbow joint function at the last follow-up was evaluated according to the Flynn criteria, and the breakdown of functional and cosmetic components is shown in Table 1. The results were as follows: 19 cases (86.4%) were excellent; 3 cases (13.6%) were good; 0 cases were poor. Among them, there was no significant difference in the range of motion of the elbow joint compared with the healthy side, and the daily living function recovered well.

**Table 1:** Breakdown of Flynn criteria scores at last follow-up.

Outcome component	Excellent (*n*, %)	Good (*n*, %)	Poor (*n*, %)
Functional (Flexion-Extension & Symmetry)	18 (81.8%)	4 (18.2%)	0 (0%)
Cosmetic	19 (86.4%)	3 (13.6%)	0 (0%)
Combined Excellent/Good Rate	-	-	22 (100%)

### 3.2 Demonstrative cases

**Figure 1:**
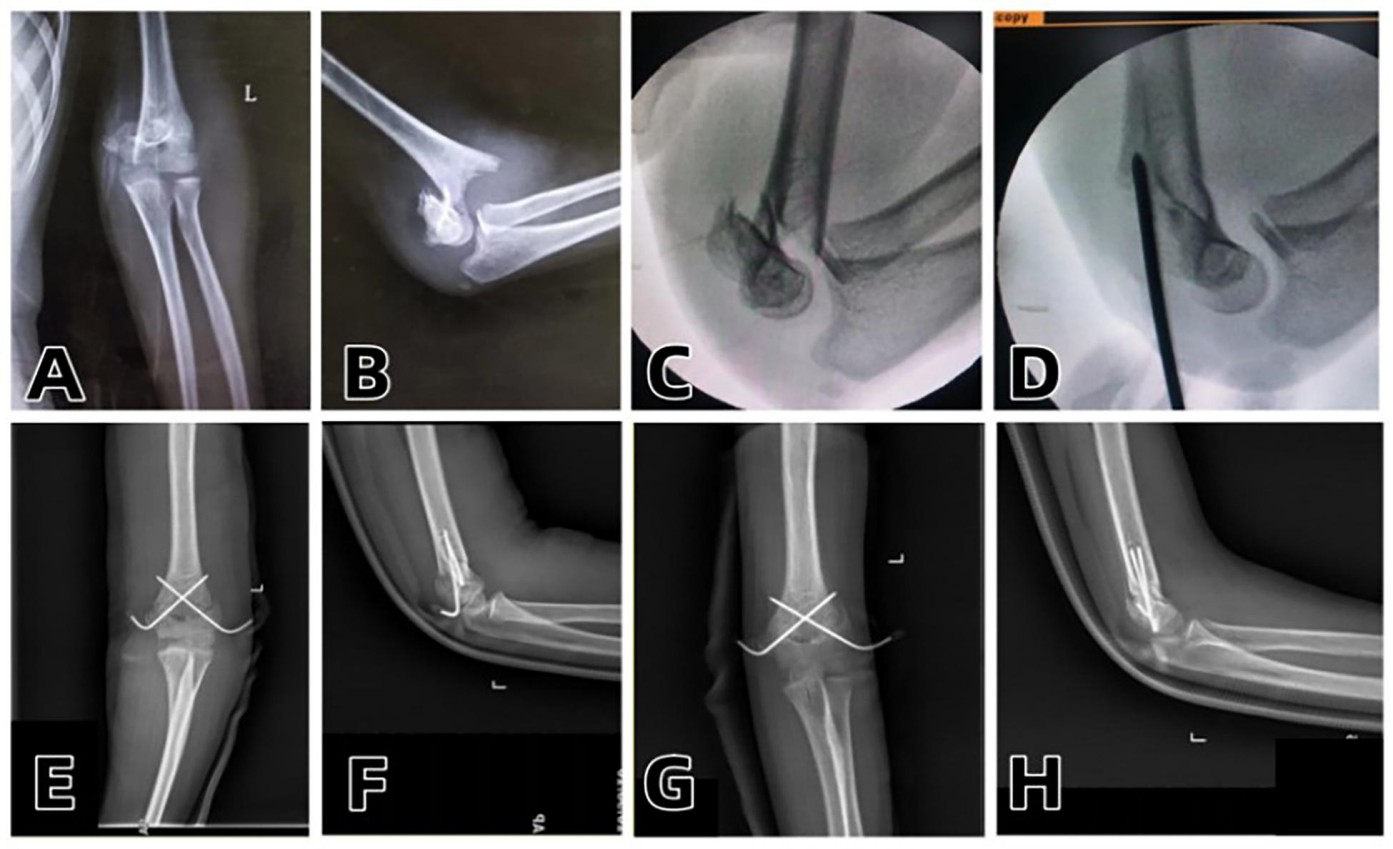
**(A,B)** Preoperative anteroposterior and lateral radiographs of the elbow joint; **(C)** Intraoperative failure of manual reduction; **(D)** Successful intraoperative reduction with a single Kirschner wire prying; **(E,F)** Intraoperative anteroposterior and lateral radiographs after inserting one Kirschner wire on each of the medial and lateral sides and applying plaster fixation; **(G,H)** Reexamination at 1 month after surgery showing good fracture healing.

**Figure 2:**
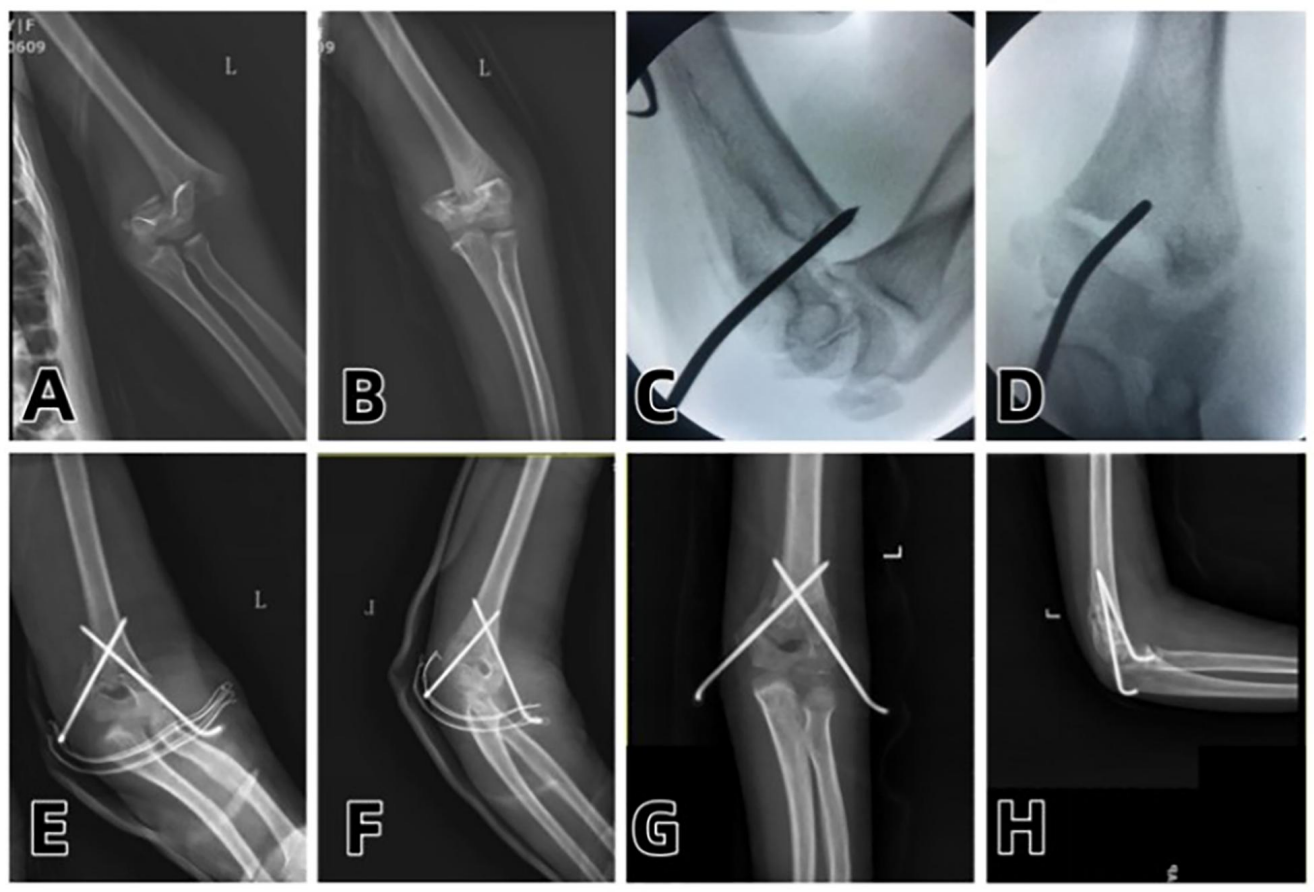
**(A,B)** Preoperative anteroposterior and lateral radiographs of the elbow joint; **(C)** Intraoperative failure of manual reduction; **(D)** Successful intraoperative reduction with a single Kirschner wire prying; **(E,F)** Intraoperative anteroposterior and lateral radiographs after inserting one Kirschner wire on each of the medial and lateral sides and applying plaster fixation; **(G,H)** Reexamination at 1 month after surgery showing good fracture healing.

**Figure 3:**
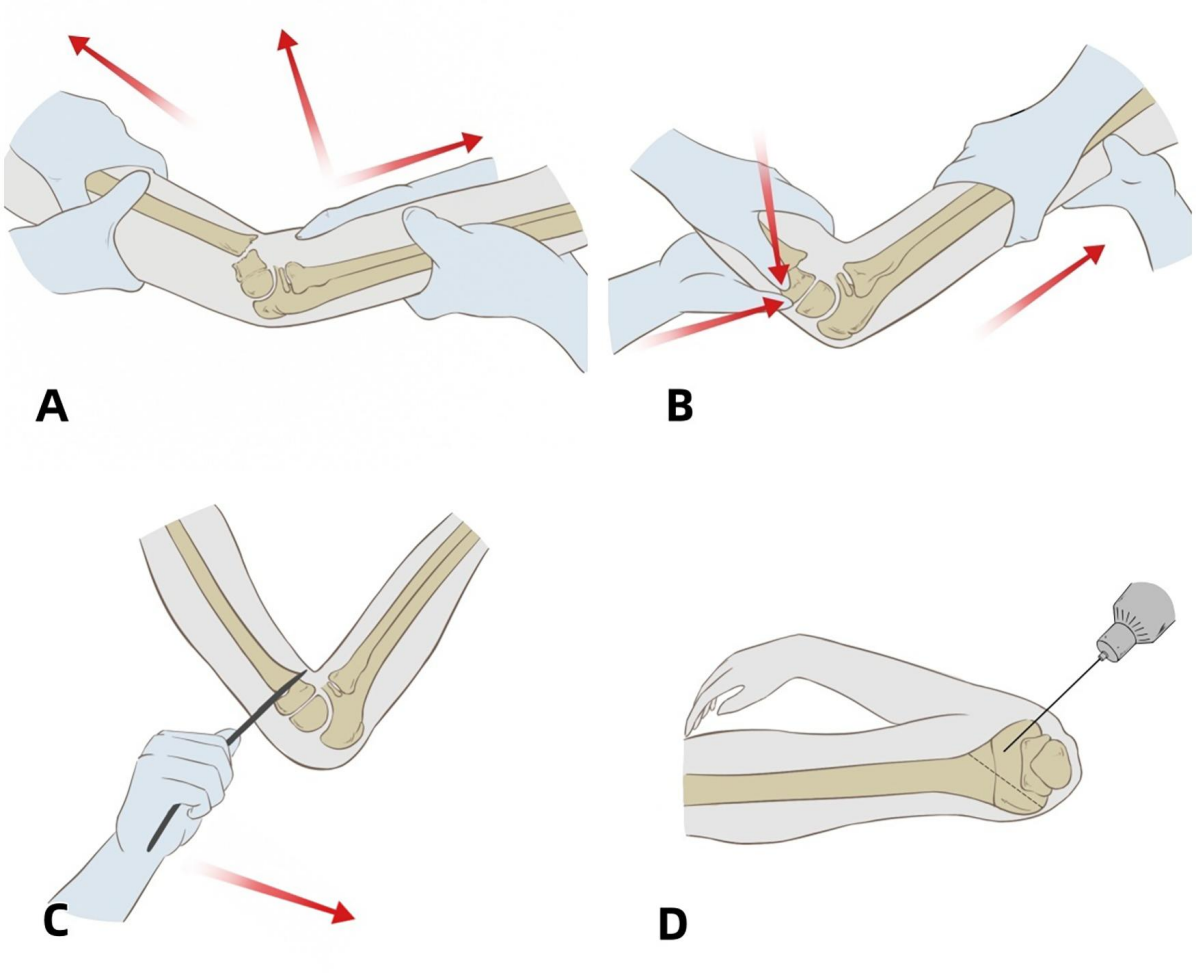
**(A)** intraoperative traction process; **(B)**: intraoperative manual reduction; **(C)** intraoperative reduction with a single Kirschner wire prying; **(D)** Kirschner wire fixation of the fracture.

Two typical cases with complete pre-operative, intra-operative and post-operative imaging data were selected for detailed display in this study. The failure of manual reduction, the process of Kirschner wire prying-assisted reduction and the post-operative imaging comparison were presented respectively (see Figures 1–3).

Surgical Techniques:

Preoperative Preparation:

Anesthesia: General anesthesia is usually adopted to ensure the child undergoes the operation in a painless state.
Body Position: The child lies in a supine position, with the shoulder of the affected limb close to the edge of the operating table, and the affected limb is abducted and pronated to facilitate operation.
Disinfection and Draping: Thoroughly disinfect the affected limb and spread sterile drapes to ensure the sterility of the surgical area.
Equipment Preparation: Place the C-arm machine beside the operating table for intraoperative fluoroscopy to ensure the effect of reduction and fixation.

Closed Reduction Manipulation: The surgeon stands at the distal end of the affected limb, holds the proximal part of the forearm of the affected limb, with the elbow joint at 30–45°. The assistant stands at the head side of the patient's shoulder, holds the upper arm of the affected limb, and performs counter-traction under fluoroscopy to align the fracture and correct rotation. Then the assistant moves to stand under the affected armpit and holds the forearm, slowly flexes the elbow joint to 90°. The surgeon holds the distal end of the humerus with both hands, and presses the posterior parts of the medial and lateral condyles respectively with the thumbs of both hands at the posterior side of the elbow joint. The assistant holds the forearm to perform counter-traction on the affected limb, adjusts the ulnar deviation, radial deviation and anterior-posterior angulation of the fracture. The assistant slowly flexes the elbow and externally rotates the affected limb until the elbow joint is extremely flexed (120° or more), and observes the lateral reduction under fluoroscopy. The assistant maintains extreme flexion with both hands to prevent re-displacement of the fracture end.

Prying Reduction: If the manual reduction is not ideal, prying reduction can be performed under fluoroscopic guidance. Insert the tip of the Kirschner wire into the fracture end percutaneously from the anterior or posterior side, and use the lever principle to pry the distal or proximal end of the fracture for reduction under fluoroscopy. The surgeon adjusts the needle insertion angle and depth to directly control the movement of the fracture fragment and correct rotational displacement or complex misalignment. If necessary, bend and shape the tip of the Kirschner wire to facilitate reduction.

Kirschner Wire Fixation: After the reduction is completed, use 2–3 Kirschner wires with a diameter of 2.0–2.5 mm. Insert the wires respectively from the apex of the lateral epicondyle of the humerus at a 45° angle to the longitudinal axis of the humeral shaft, and 5 mm in front of the medial epicondyle of the humerus, avoiding the ulnar nerve groove. The medial Kirschner wire penetrates the lateral cortical bone of the proximal end and crosses with the lateral Kirschner wire at 1 cm proximal to the fracture line, forming a stable “X”-shaped fixation.

Postoperative Management: Immediately after the operation, apply a plaster fixation with the elbow flexed at 45° for 4 weeks. When the elbow is flexed at 45°, the tension of the biceps brachii is reduced by 40%, which reduces the risk of compartment syndrome and is beneficial for subsequent functional exercises. Conduct regular follow-up after surgery, perform x-ray reexaminations, monitor the fixation status of the Kirschner wires, and conduct regular pin tract care. During the recovery period, avoid intense elbow activities to prevent unstable reduction. After removing the plaster slab, the patient begins to perform moderate elbow activity training to gradually restore joint flexibility and prevent joint stiffness.

## 4 Discussion

This study retrospectively analyzed 24 cases of Gartland Type IV supracondylar humeral fractures in children. Closed reduction assisted by Kirschner wire prying achieved a closed reduction success rate of 91.7%, and all cases obtained good fracture healing and elbow joint function recovery after surgery. This indicates that for Gartland Type IV cases with failed manual reduction, closed Kirschner wire prying technique is a safe and effective minimally invasive alternative, with good clinical promotion prospects.

Due to the complete rupture of the periosteum in Gartland Type IV fractures, the proximal and distal fracture ends are unstable in both the coronal and sagittal planes. Traditional traction reduction is prone to reduction failure or re-displacement after reduction (5). According to literature reports, the open reduction rate of such fractures is as high as 15%–30% (6). Open reduction and internal fixation (ORIF) is a traditional method for the treatment of Gartland Type IV fractures, which is suitable for cases with difficult reduction or severe displacement. Literature has repeatedly mentioned that ORIF technology can accurately restore fracture alignment and reduce the risk of re-displacement after reduction (7). However, open surgery exposes the fracture end, which may lead to soft tissue dissection and neurovascular injury, with a long post-operative recovery period and risks of joint stiffness and infection. In addition, the incidence of complications of ORIF in pediatric patients increases with age, and problems such as joint stiffness and scar formation become more prominent (8).

Closed reduction combined with Kirschner wire fixation is one of the conventional treatment options for Gartland Type IV fractures, especially suitable for pediatric patients without obvious neurovascular injury. In the traditional closed reduction process, fractures that are difficult to reduce often require auxiliary traction or manual prying, and the surgical success rate is closely related to the surgeon's experience (9). Compared with traditional manual reduction, the closed Kirschner wire prying reduction technique uses fluoroscopic guidance and the Kirschner wire as a lever to finely adjust the distal end of the fracture, resulting in a higher reduction success rate and lower complication rate (10). This study shows that the success rate of closed Kirschner wire prying reduction is as high as 91.7%. Compared with other surgical methods, it causes less trauma, has less intraoperative blood loss, and allows faster post-operative recovery, which can better avoid joint stiffness and poor reduction.

The “milking” technique is one of the simple and most commonly used closed reduction methods for supracondylar humeral fractures, suitable for simple fracture types such as Gartland Type II fractures. After restoring the limb length through longitudinal traction, the surgeon holds the proximal end of the fracture with both hands, and uses the thumbs and index fingers to simulate the pushing action of “milking”. The pushing action is performed along the longitudinal direction of the fracture end on the palmar side of the elbow joint to release the soft tissue impaction at the fracture end, achieve indirect reduction, and gradually correct the displacement in the coronal and sagittal planes, while reducing direct stimulation to the fracture end (11). The joystick technique is a hot technique for assisting the reduction of Gartland Type III fractures (12). It usually uses 1 Kirschner wire to manipulate the fracture end for auxiliary reduction. By pushing the Kirschner wire and manipulating the proximal or distal end of the fracture, rotational displacement is corrected and coronal reduction is achieved (13). A study used 2 Kirschner wires inserted through the olecranon of the ulna and the distal 1/3 of the humerus respectively to manipulate the two fracture ends, allowing the two fracture ends to “actively” align with each other under manipulation instead of one end passively approaching the other, so as to correct multidirectional displacement and achieve reduction of multidirectionally unstable fractures (10). The main disadvantage of the joystick technique is its high technical requirements for operation (14). It is necessary to insert a Kirschner wire in advance as a joystick, which not only increases additional trauma and the potential risk of neurovascular injury, but also may interfere with the placement of the final fixation wire due to conflicts in the needle insertion site.

The closed Kirschner wire prying reduction technique is suitable for Gartland Type III and IV fractures (with complete displacement or obvious rotational displacement), especially for cases with failed closed reduction or those requiring correction of displacement in multiple directions at the same time. Through the synergistic effect of swinging, traction and squeezing, the Kirschner wire lever prying can accurately control rotational displacement, comprehensively correct the displacement of the fracture end, improve the reduction quality and achieve better treatment effects (15).

In recent years, with the development of minimally invasive surgical technology, methods such as nail-plate fixation and percutaneous plate internal fixation have been proposed for the treatment of Gartland Type IV fractures. Minimally invasive nail-plate fixation technology restores fracture alignment through smaller incisions and precise implantation processes, which can minimize post-operative complications. However, this method also faces high surgical difficulty and requires relatively precise imaging guidance to ensure the stability of fixation (16). Compared with these technologies, closed Kirschner wire prying reduction does not require additional incisions. With the help of image guidance, the Kirschner wire is used as a lever tool to complete fine reduction without exposing the fracture end, and the direction of the distal fracture is dynamically adjusted during the operation (10). Among the 24 cases in this group, no intraoperative neurovascular injury occurred, and only 1 case had pin tract infection after surgery, which improved after dressing change, with no obvious malunion. It has the following advantages: (1) Minimally invasive and efficient: Based on the principle of lever mechanics, it achieves easy and accurate reduction, converts complex anatomical reduction into controllable mechanical regulation, effectively avoids soft tissue injury caused by repeated operations, and fully conforms to the concept of modern minimally invasive orthopedic treatment. The trauma is reduced by more than 60% compared with traditional open reduction and internal fixation (17), (2) Simple and convenient operation: Only basic instruments such as prying needles are needed to complete reduction and fixation. Its dual functions (reduction tool + temporary fixation) optimize the surgical process by 30%. In this study, the average operation time was shortened to 42.5 ± 9.3 min, which is particularly suitable for pediatric patients, significantly reducing the risk of general anesthesia and the incidence of complications, (3) Blood supply protection: Percutaneous prying technology maximizes the maintenance of blood supply at the fracture end. Clinical observations in this study showed that the time of callus formation was advanced by 5–7 days after surgery, and the excellent and good rate of functional recovery reached 100% (18), (4) Infection prevention and control: It avoids exposure of surgical incisions, significantly reducing the risk of deep infection (19), (5) Economical and convenient: The internal implant Kirschner wire can be safely removed in the outpatient department, eliminating the need for secondary inpatient surgery, reducing the overall treatment cost, and significantly alleviating the economic burden of patients and the consumption of medical resources; (6) Short learning curve: The operation is relatively simple for surgeons with certain experience.

This study has certain limitations. The sample size is small, and there is a lack of a control group, so statistical comparison cannot be carried out. Secondly, Gartland Type IV is an intraoperative diagnosis, which is difficult to fully identify preoperatively, and the surgeon needs to have certain intraoperative judgment ability. Thirdly, Kirschner wire prying operation needs to be completed with the cooperation of C-arm, which has certain requirements for the technology and experience of the operating personnel. Additionally, due to the tight timeline of the study (last patient recruitment in May 2025 and manuscript submission in December 2025), long-term complications such as avascular necrosis, which may manifest beyond 6 months of follow-up, could not be fully evaluated in the most recent cases. In the future, multi-center joint studies can be conducted to expand the sample size and set up a control group with traditional open reduction to further verify the stability, reduction accuracy and long-term elbow joint function recovery of this technology.

## 5 Conclusion

The closed Kirschner wire prying-assisted reduction technique shows a high closed reduction success rate and good functional recovery effect in the treatment of Gartland Type IV supracondylar humeral fractures. It has the advantages of small trauma, simple operation and high safety, and offers a viable alternative to open reduction, which is a minimally invasive reduction strategy worthy of clinical promotion.

## Data Availability

The original contributions presented in the study are included in the article, further inquiries can be directed to the corresponding authors.
